# Response time and eye tracking datasets for activities demanding varying cognitive load

**DOI:** 10.1016/j.dib.2020.106389

**Published:** 2020-10-08

**Authors:** Prarthana Pillai, Prathamesh Ayare, Balakumar Balasingam, Kevin Milne, Francesco Biondi

**Affiliations:** a Department of Electrical and Computer Engineering, University of Windsor, 401 Sunset Avenue, Windsor, ON, N9G 3P4, Canada; b Faculty of Human Kinetics, University of Windsor, 401 Sunset Avenue, Windsor, ON N9G 3P4, Canada

**Keywords:** Cognitive load detection, Eye-tracking, Pupil dilation, Human-computer interface, Detection response task (DRT), Psychological signals, Detection, Signal processing, Machine learning

## Abstract

The dataset contains the following three measures that are widely used to determine cognitive load in humans: Detection Response Task - response time, pupil diameter, and eye gaze. These measures were recorded from 28 participants while they underwent tasks that are designed to permeate three different cognitive difficulty levels. The dataset will be useful to those researchers who seek to employ low cost, non-invasive sensors to detect cognitive load in humans and to develop algorithms for human-system automation. One such application is found in Advanced Driver Assistance Systems where eye-trackers are employed to monitor the alertness of the drivers. The dataset would also be helpful to researchers who are interested in employing machine learning algorithms to develop predictive models of humans for applications in human-machine system automation. The data is collected by the authors at the Department of Electrical & Computer Engineering in collaboration with the Faculty of Human Kinetics at the University of Windsor under the guidance of their Research Ethics Board.

**Specifications Table**

**Table:** 

Subject	Human Factors and Ergonomics
Specific subject area	Cognitive load detection based on eye-tracking and response time
Type of data	Table Excel workbook
How data were acquired	Cognitive load experienced by human subjects were artificially changed (using delayed digital recall task). Instruments: Gazepoint GP3 Eye-tracker, Vibrotactile Detection Response Task (DRT) Make and model and of the instruments used: Gazepoint GP3, Red Scientific Limited
Data format	Raw
Parameters for data collection	Cognitive load is the main parameter that differentiates recorded data and four types of cognitive load measures (performance based, physiological, behavioural, subjective) were recorded.
Description of data collection	The dataset contains multiple measures of cognitive load collected from 28 participants while they performed tasks of varying cognitive difficulty. The primary task was administered using the delayed digital recall task (also called the N-back task) where an audio stimuli is provided and participants are required to verbally recall them; the difficulty increased with the delay, i.e., difficulty of recalling 0-back, 1-back, and 2-back increases in that order. A DRT, where participants are required to respond to a vibration stimulus by pressing a switch, is used as a behavioural measure of cognitive load. The DRT is also used as a secondary task, i.e., the experiment was repeated with and without DRT. Eye-tracking measures, such as pupil diameter and eye-gaze are collected live during the entire experiment. The N-back data allows to compute the accuracy of digital recall task and use it as a performance measure of cognitive load. In addition to this, NASA Task Load Index (NASA TLX) scores were collected at the end of each experiment as a self-reported measure of cognitive load.
Data source location	Institution: University of Windsor City/Town/Region: Windsor, Ontario Country: Canada Latitude and longitude (and GPS coordinates, if possible) for collected samples/data: 42.2997° N, 83.0617° W
Data accessibility	Repository name: Mendeley Data Data identification number: 10.17632/dp8g983t38.1Direct URL to data: https://data.mendeley.com/datasets/dp8g983t38/draft?a=1a80ae60–6591–4a0a-b20a-4be2de002df3
Related research article	F. N. Biondi, B. Balasingam, and P. Ayare, On the cost of detection response task performance on cognitive load, Human factors (2020).https://doi.org/10.1177/0018720820931628

## Value of the Data

• The data provides several measures, taken simultaneously, while the participants underwent specific activities that are designed to require varying levels of cognitive load.
• This data can be used to train machine learning algorithms that employ the same physiological and behavioural measures in order to develop solutions that require to predict cognitive load in real time.
• The data can be used for in-depth understanding of the results presented in the related research article.
• The data will be useful to those researchers who seek to understand the effectiveness of low-cost sensors in order to estimate cognitive load experienced by humans.
• The data can be used to test the applicability of predictive modeling algorithms and machine learning as a way to classifying cognitive load.

## 1 Data Description

### 1.1 General introduction

System automation with humans in the loop is one of the biggest challenges in the 21st century. With technological elements embedded in all aspects of our everyday life, it is essential to measure the level of fluctuating cognitive load experienced by humans, to ensure safety during the operation of automated systems. For instance, in partially automated driving environments, inaccurate estimation of cognitive load may result in greater crash risk due to driver distraction [2]. Hence, non-invasive metrics that can be used as reliable indicators of cognitive load must be developed for safe adoption in human-machine systems.

The International Organization for Standardization(ISO 17488:2016) provides a paradigm known as Detection Response Task (DRT) that is intended for assessing ``the attentional effects of cognitive load on attention for secondary tasks involving interaction with visual-manual, voice-based or haptic interfaces" [3]. It quantifies the dynamic changes in operators' cognitive load resulting from attentional allocation to one or more tasks at hand. In driving environments, as in [4], response times to DRT stimuli increases with the driving demand and with the difficulty of the cognitive auditory task. In addition to DRT, studies also show changes in pupil diameter under conditions of varying cognitive load [5]. Thus, these two non-intrusive metrics may be used to accurately determine cognitive load experienced by humans. The objective of this study is to demonstrate and develop reliable cognitive load detection metrics based on pupil dilation and response time data.

**Fig. 1:**
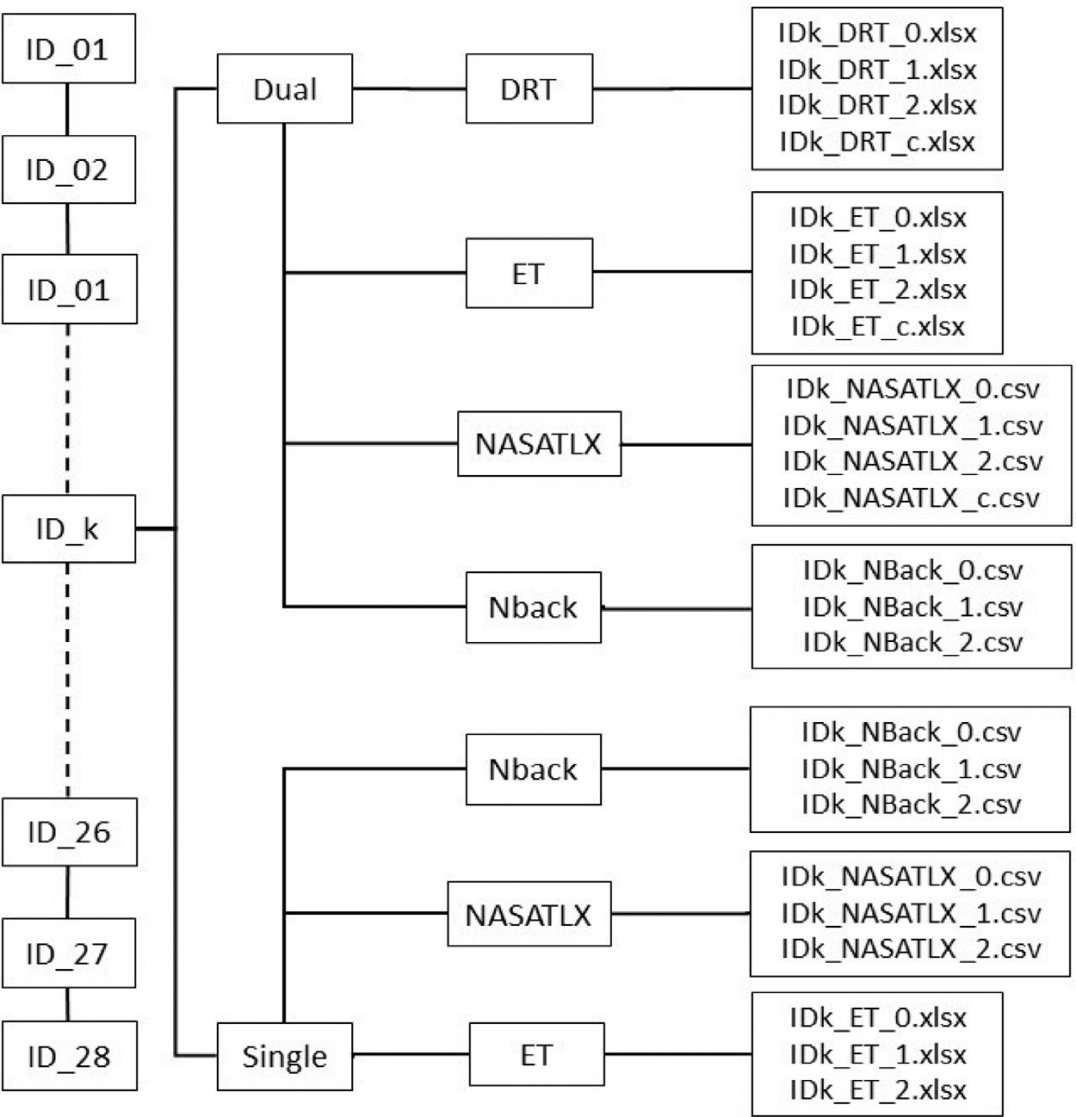
Contents of the dataset. The data contains 28 folders for 28 participants. Each folder is divided into two folders named Dual and Single. The NASA TLX, Nback and ET data are stored in each of these folders. The DRT data is available in the Dual folder.

This article presents the datasets used in [1]. This data was collected from 28 participants. Each participant underwent seven experimental conditions in two difficulty levels: Dual (with DRT) and Single (without DRT). Dual experiment had the following four difficulty conditions: c (Control), 0-back, 1-back, 2-back, where the difficulty increases in that order; and the Single experiment had the following three difficulty conditions: 0-back, 1-back, 2-back. Each experimental condition lasted approximately 5 min during which the participants were always instructed to keep their gaze fixed on the display. A desktop mounted eye-tracker was used record the pupil diameter information in pixels and response times in milliseconds was recorded for stimulus from a vibrotactile version-based DRT. For each condition, the responses to the delayed digital recall task called n-back task were also recorded in separate excel files. An electronic version of the NASA Task Load Index (NASA TLX) questionnaire was administered after each condition. Fig. 1 summarizes the arrangement of files for all participants in the dataset for different conditions; Control, 0-Back, 1-Back, and 2-Back denoted as c, 0, 1, and 2, respectively.

**Table 1:** Contents of DRT data. Each excel file in the DRT folder contains the following data.

Column Name	Column Number	Description
Trial Start	1	Starting time in the format: Minute.Second.Millisecond
Date	2	Trial date
Time	3	The time elapsed (in milli-seconds) since the last system initialization or calibration.
Trial	4	Trial/vibration counter
Clicks	5	Participant response to the vibration - `0′ for no response
Response time	6	The response time (in milliseconds) of the participant to the vibration stimulus - `−1′ for no response.

**Fig. 2:**
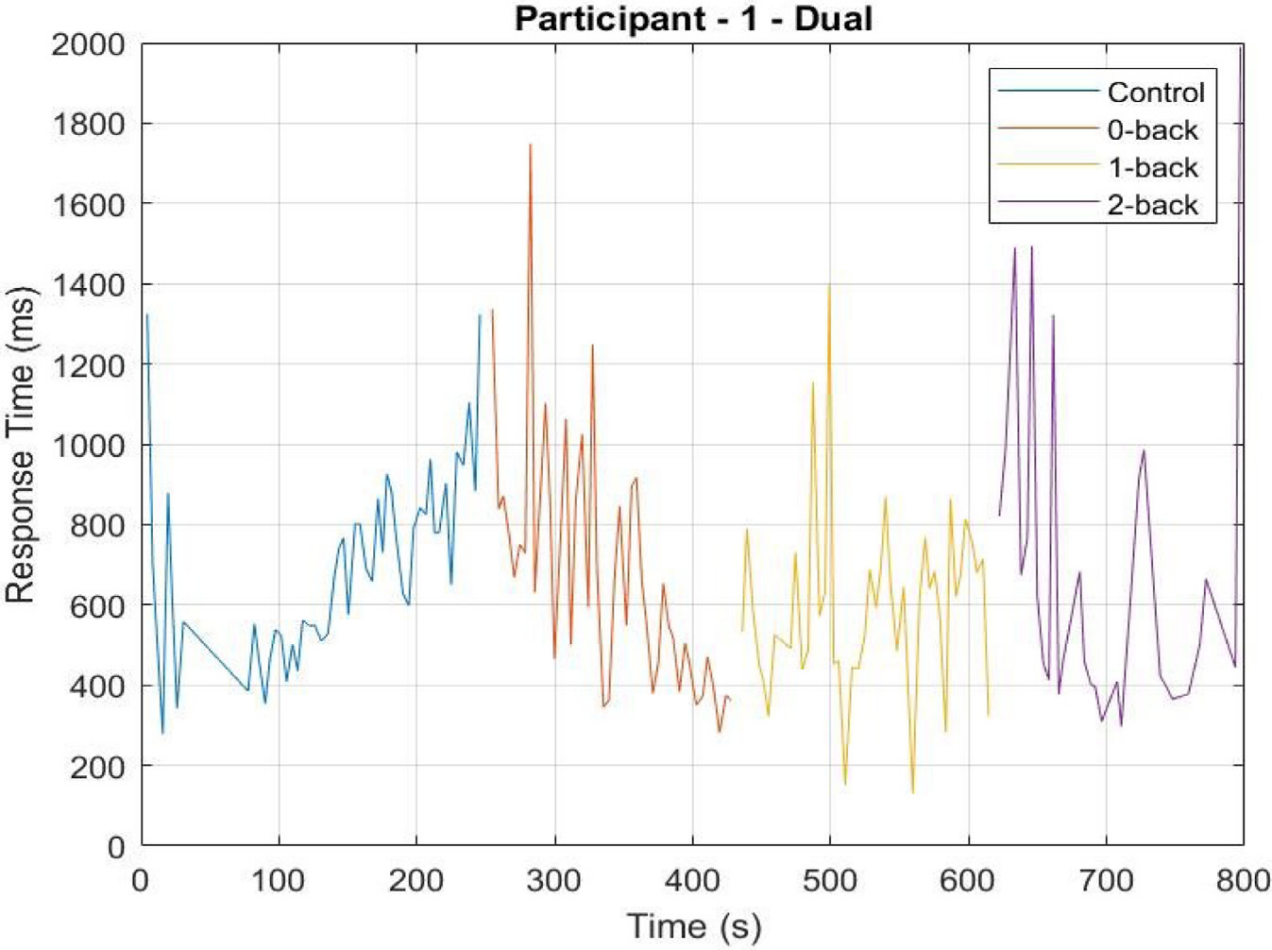
Sample response time for participant 01. The Response Time vs. Experiment Time is shown. The MATLAB program ‘DRT.m’ (available in the parent folder) will generate the above plot.

### 1.2 DRT data

The DRT data is stored in files named in the following format: “(participantID)_DRT_(condition).xlsx” where participantID varies from ‘ID01’ to ‘ID28’ and the condition can be ‘c’,‘0’,‘1’,‘2’ (see Fig. 1 for explanation). Each of these files have six columns; see Table 1 for detailed description of these six columns. Fig. 2 shows a sample plot of ‘Response time vs. Time’ for participant 01 generated using the MATLAB file DRT.m in the source link given in specifications table. Time in the axis is aligned for visualization purposes.

### 1.3 Eye tracker data

**Fig. 3:**
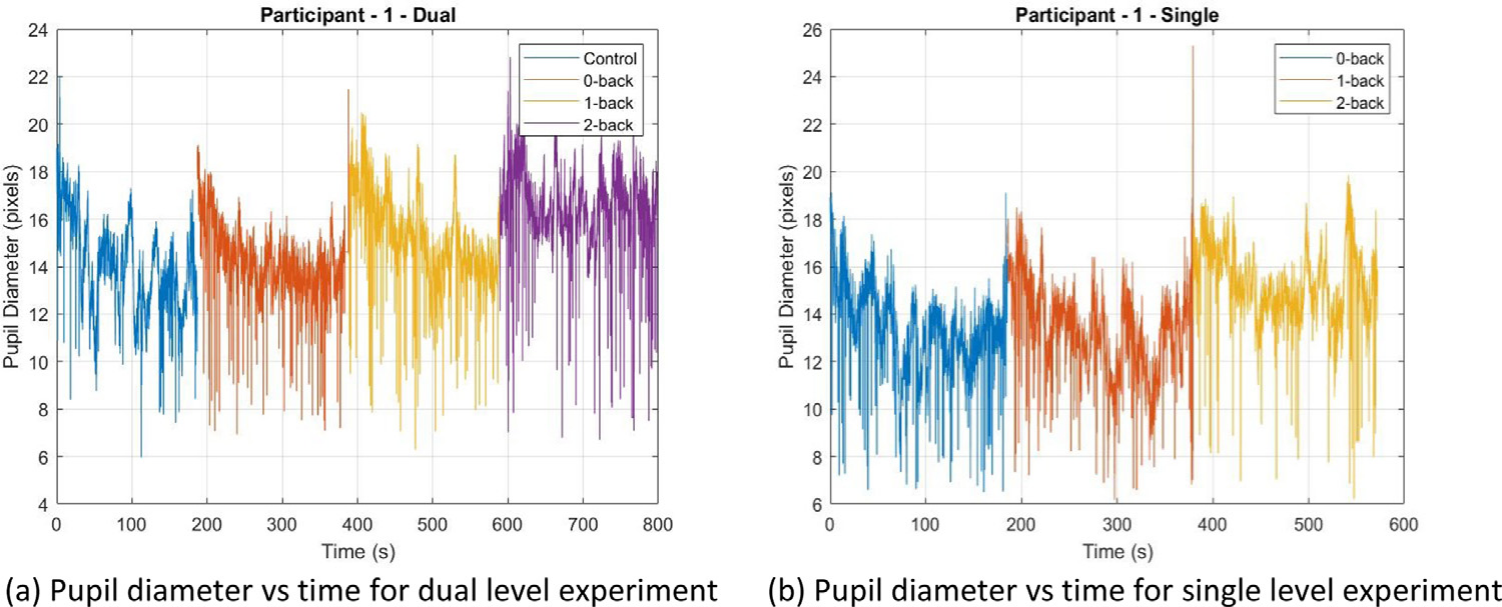
Sample left pupil diameter data for participant 01. The Pupil Diameter vs. Experiment Time is shown. The MATLAB program ‘PD.m’ (available in the parent folder) will generate the above plot.

The pupil dilation data was measured by the Gazepoint GP3 Eye-tracker [6] for each condition of both levels of the experiment and saved in 7 excel files that are named in the following format: “(participantID)_ET_(condition).xlsx” where participant ID varies from ‘ID01’ to ‘ID28’ and condition can be ‘c’,‘0’,‘1’,‘2’ (see Fig. 1 for explanation). Each file contains pupil data measured for approximately 5 min and Table 2 provides a description of important columns in this excel data file. Fig. 3 shows sample plot of pupil diameter of the left eye vs time for both conditions: Dual and Single for participant 01 generated using the MATLAB file PD.m saved in the source link.

**Table 2:** Contents of pupil data from Gazepoint GP3 Eye-tracker [6]. Each excel file in the ET folder contains the following data.

Column name	Column number	Description
Media ID and name	1, 2	ID and name of media
Counter	3	This variable is incremented by 1 for each data record sent by the server.
Time	4	The time elapsed in seconds since the last system initialization or calibration. Usually between 0 and 350 milli-seconds
Timetick	5	A signed 64-bit integer which indicates the number of CPU time ticks.
FPOGX, FPOGY	6, 7	The X- and Y-coordinates of the Fixation Point of Gaze (FPOG), as a fraction of the screen size. (0,0) is top left, (0.5,0.5) is the screen center, and (1.0,1.0) is bottom right.
FPOGS, FPOGD	8, 9	The starting time and duration of the fixation POG in seconds since the system initialization or calibration.
FPOGID	10	The fixation POG ID number.
FPOGV	11	The valid flag with value of 1 if the FPOG data is valid, and 0 if it is not.
BPOGX, BPOGY	12, 13	The X- and Y-coordinates of the best eye POG which is the average of the left eye and right eye POG if both are available, or if not, then the value of either the left or right eye, depending on which one is valid.
BPOGV	14	The valid flag with value of 1 if the BPOG data is valid, and 0 if it is not.
CX, CY, CS	15, 16, 17	The X-coordinate, Y-coordinate, and state of the mouse cursor.
USER	18	A custom data field that may be set by the user to contain any desired information such as synchronization markers.
LPCX, LPCY	19, 20	The X and Y-coordinates of the left eye pupil in the camera image, as a fraction of the camera image size.
LPD	21	The diameter of the left eye pupil in pixels.
LPS	22	The scale factor of the left eye pupil (unitless). Value equals 1 at calibration depth, is less than 1 when user is closer to the eye tracker and greater than 1 when user is further away.
LPV	23	The valid flag with value of 1 if the data is valid, and 0 if it is not.
RPCX, RPCY	24, 25	The X- and Y-coordinates of the right eye pupil in the camera image, as a fraction of the camera image size.
RPD	26	The diameter of the right eye pupil in pixels.
RPS	27	The scale factor of the right eye pupil (unitless). Value equals 1 at calibration depth, is less than 1 when user is closer to the eye tracker and greater than 1 when user is further away.
RPV	28	The valid flag with value of 1 if the pupil data of the right eye is valid, and 0 if it is not.
BKID, BKDUR, BKMIN	29,30,31	blink counter, blink duration for the last blink and the average blink rate over the last minute, respectively.

**Fig. 4:**
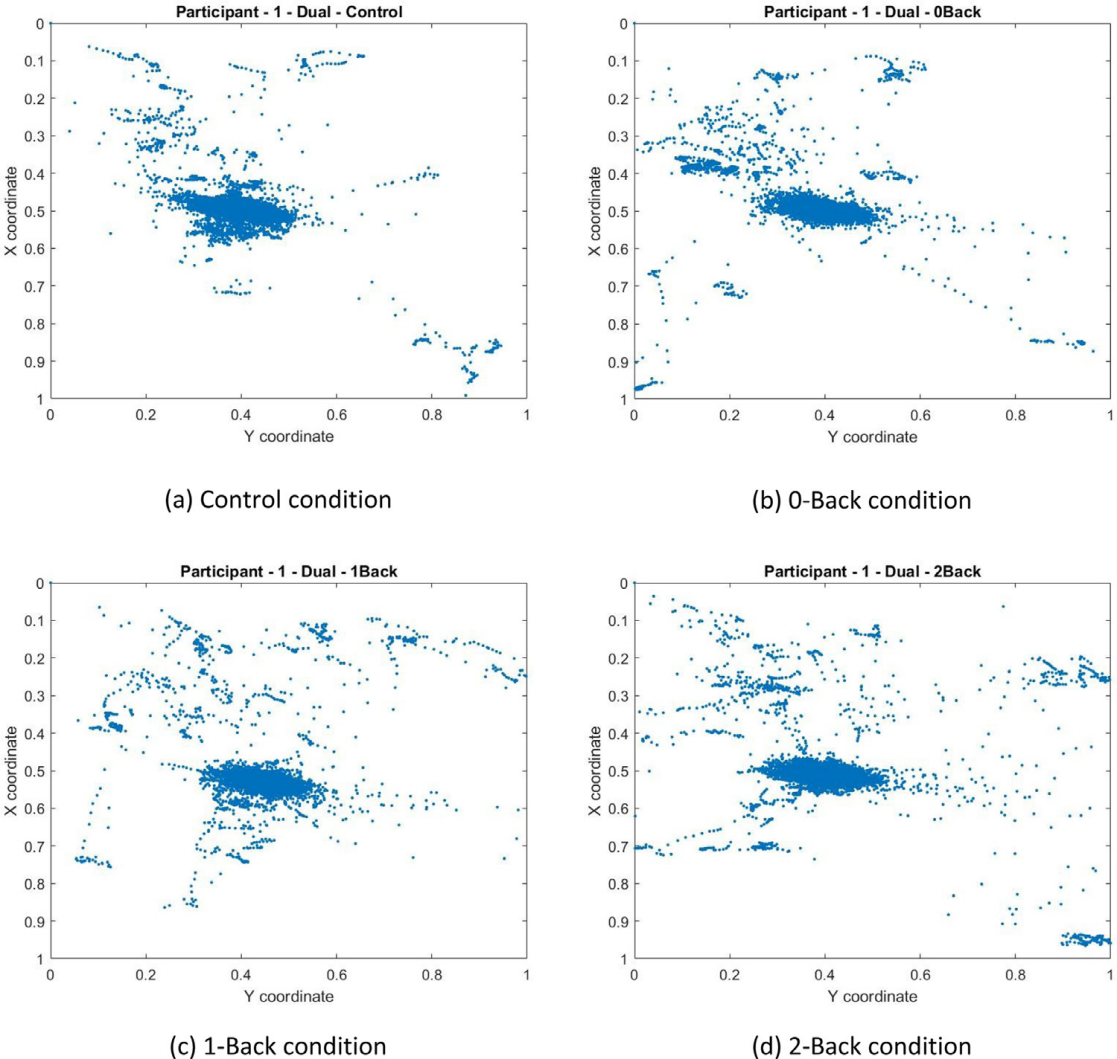
Sample eye-gaze data for participant 01. The position of the eye-gaze on the screen is indicated using a ‘●’. The eye-gaze is measured at 60 Hz resulting in around 18,000 eye-gaze points for each experiment which took approximately 5 min. The MATLAB program ‘GAZE.m’ (available in the parent folder) will generate the above plot.

The Gazepoint GP3 tracker camera also captures the various eye positions for each target point which are then mapped to the corresponding X and Y gaze coordinates obtained in columns 6 and 7 of the excel files. From this gaze data, a plot as in Fig. 4 is created for all 4 conditions of participant 01 as a sample using the MATLAB file GAZE.m in the source link. The coordinates of the pupil data are given as a fraction of the screen size where (0,0) is top left, (0.5, 0.5) is the screen center, and (1, 1) is bottom right. This data can be useful in determining and developing eye-gaze metrics that can be used as reliable indicators of cognitive load in addition to pupil diameter and response times.

### 1.4 NASA TLX data

**Fig. 5:**
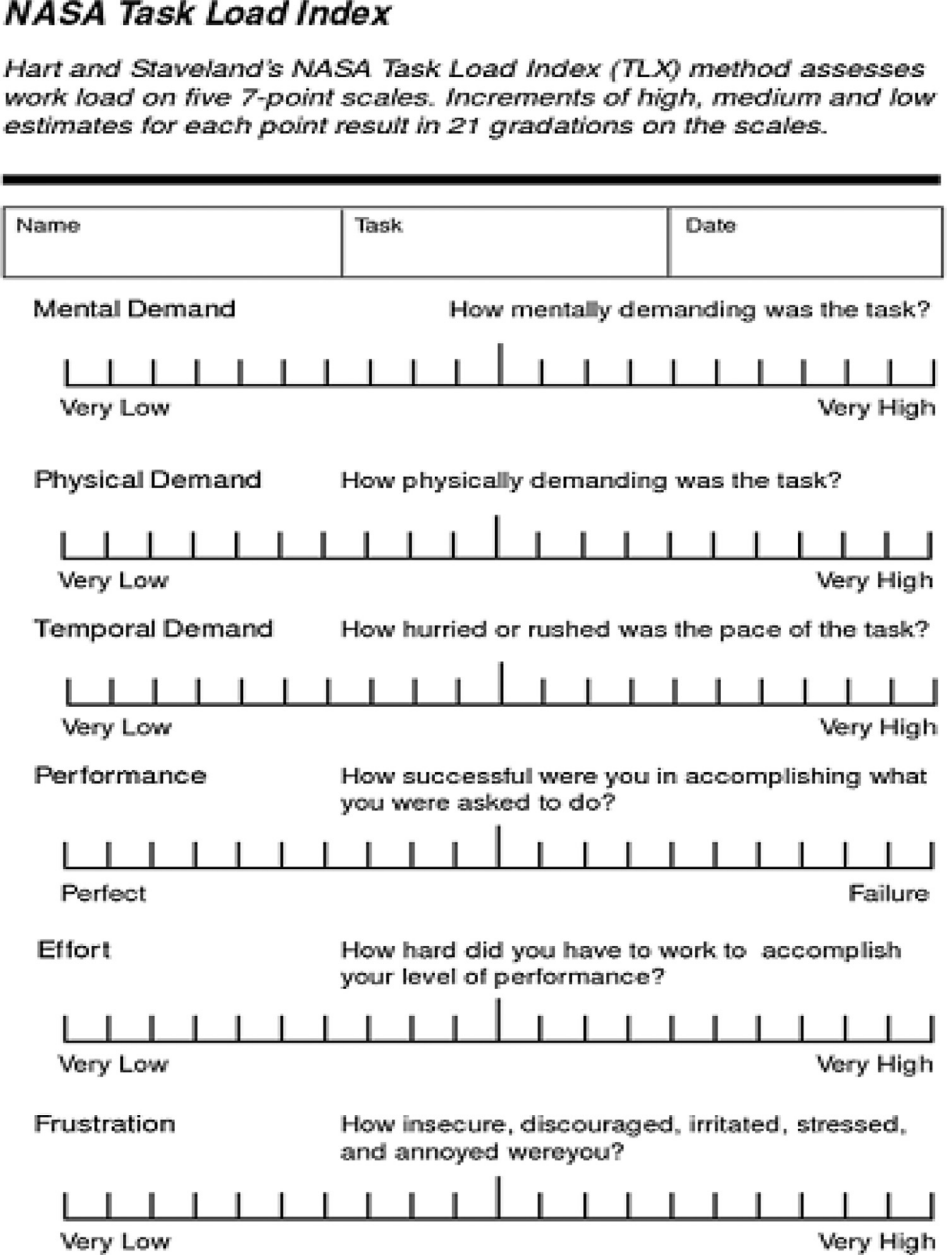
NASA TLX [7]. A picture of the NASA TLX questionnaire that was electronically administered for all 28 participants.

**Fig. 6:**
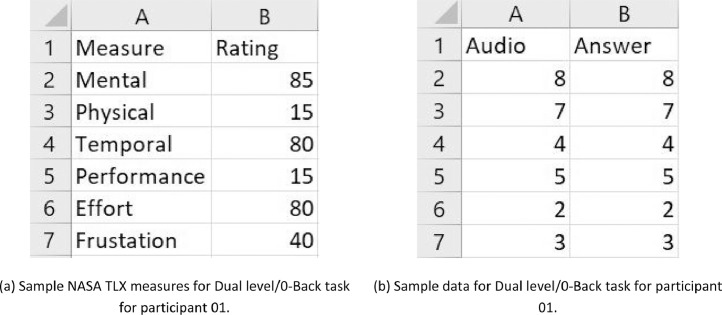
NASA TLX and N-Back Sample data for participant 01. Sample raw data is shown for NASA TLX and N-back recordings.

NASA TLX is a retrospective questionnaire that is used to measure subjective workload, see Fig. 5 [7]. The excel files in NASA TLX folder are named in the following format: “(participantID)_NASATLX_(condition).csv” where participant ID varies from ‘ID01’ to ‘ID28’ and condition can be ‘c’,‘0’,‘1’,‘2’ (see Fig. 1 for explanation). Each file contains six measures; mental, physical, temporal demands, performance, effort, and frustration rated by the participant according to their experience for each task. Fig. 6(a) is a sample of workload rating given by participant 01 for Dual level-0-Back.

### 1.5 N-Back data

Delayed digital recall task, referred to as the n-back task [8], has a serial presentation of a stimulus in the form of audio (series of numbers) spaced approximately one second apart which involves the storage and continual updating of information in working memory of the human brain. Participants listened to an audio file with series of digits, and were instructed to repeat aloud the last digit for 0-back, the penultimate digit for 1-back, and the third to last digit presented in the series for 2-back. The response from participants for each n-back task were stored in separate excel files, each with two columns of numbers: Audio (expected answer) and Answer (number repeated by the participant). These excel files are named in the following format in the Nback folder: “(participantID)_NBack_(condition).csv” where participant ID varies from ‘ID01’ to ‘ID28’ and condition can be ‘0′,‘1′,‘2′ (see Fig. 1 for explanation). The first seven observations of 0-Back task of participant 01 is displayed in Fig. 6(b) as a sample.

## 2 Experimental Design, Materials and Methods

A randomized controlled trial was conducted to determine the cognitive load (primary outcome) based on the pupil dilation and response time; with the objective of developing a new measure called SNR-Signal to Noise Ratio (secondary outcome) that is sensitive to detect multiple sources of cognitive load that would have been ignored by traditional analyses. This research complied with the American Psychological Association Code of Ethics and data was collected by the Department of Electrical & Computer Engineering in collaboration with the Faculty of Human Kinetics, approved by the Research Ethics Board (REB) at the University of Windsor.

Participants were eligible if they were between the ages of 18 and 60. 28 participants were recruited for this study and all participants had normal or corrected-to-normal vision and hearing. Informed consent was obtained from each participant and they all were given sufficient time to familiarize with the experimental setup and different conditions. All participants completed two levels: Single and Dual based on the independent measure DRT.

### 2.1 Dual experiment

A 2 task * 4 condition [i.e., c (Control), 0-back, 1-back, 2-back] within-subject experiment where participants performed n-back tasks along with DRT.

### 2.2 Single experiment

A 1 task * 3 condition [i.e., 0-back, 1-back, 2-back] within-subject experiment where participants performed the n-back task without DRT.

**Fig. 7:**
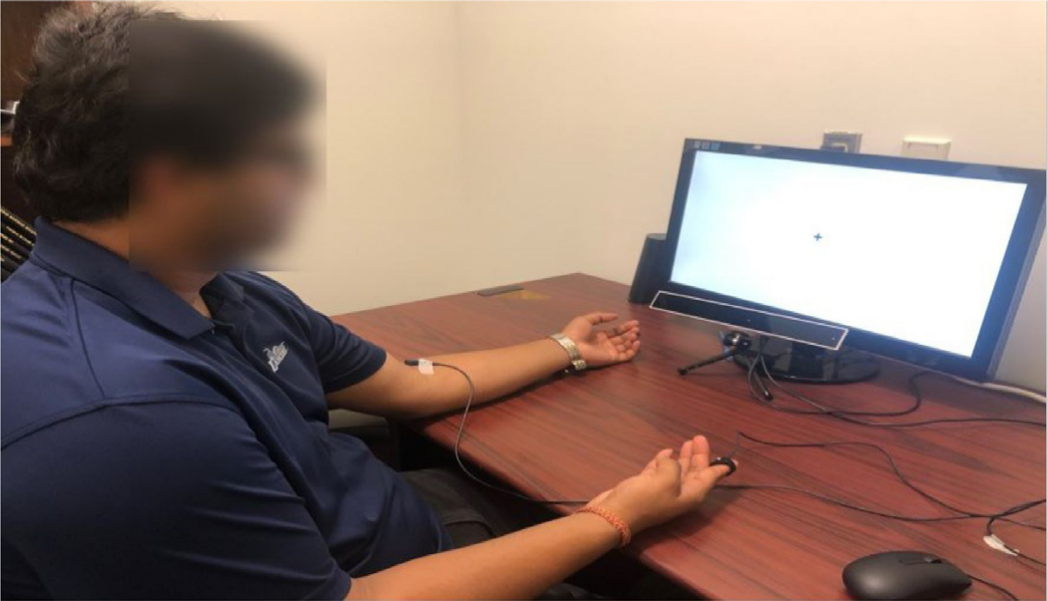
Experimental Setup. The eye-tracker is placed under the screen. The DRT stimuli is attached to the left hand of the participant and the DRT response switch is shown at the right hand of the participant. The participants are asked to always look at the ‘+’ sign shown on the screen.

The data collection phase lasted approximately 40 min. When DRT was present, participants completed 4 conditions: control (only DRT, no n-back), DRT + 0-back, DRT + 1-back, DRT + 2-back. When DRT was absent, participants completed 3 conditions with no DRT: 0-back, 1-back, 2-back. A condition with no DRT and no n-back was not considered. In total, 7 conditions were performed by each participant, counterbalanced using a Latin square table. During each condition, participants were instructed to keep their gaze fixed on the cross presented at the center of the display located approximately 30 cm away, see Fig. 7. A PC running Windows 10, with a screen resolution of 1920 × 1080 was used. Each of the seven experimental conditions lasted approximately 5 min. At the end of each condition, participants completed the NASA TLX (for a total of seven times), after which the next condition commenced.

The description about apparatus used in data collection is as follows:

### 2.3 DRT recorder

**Fig. 8:**
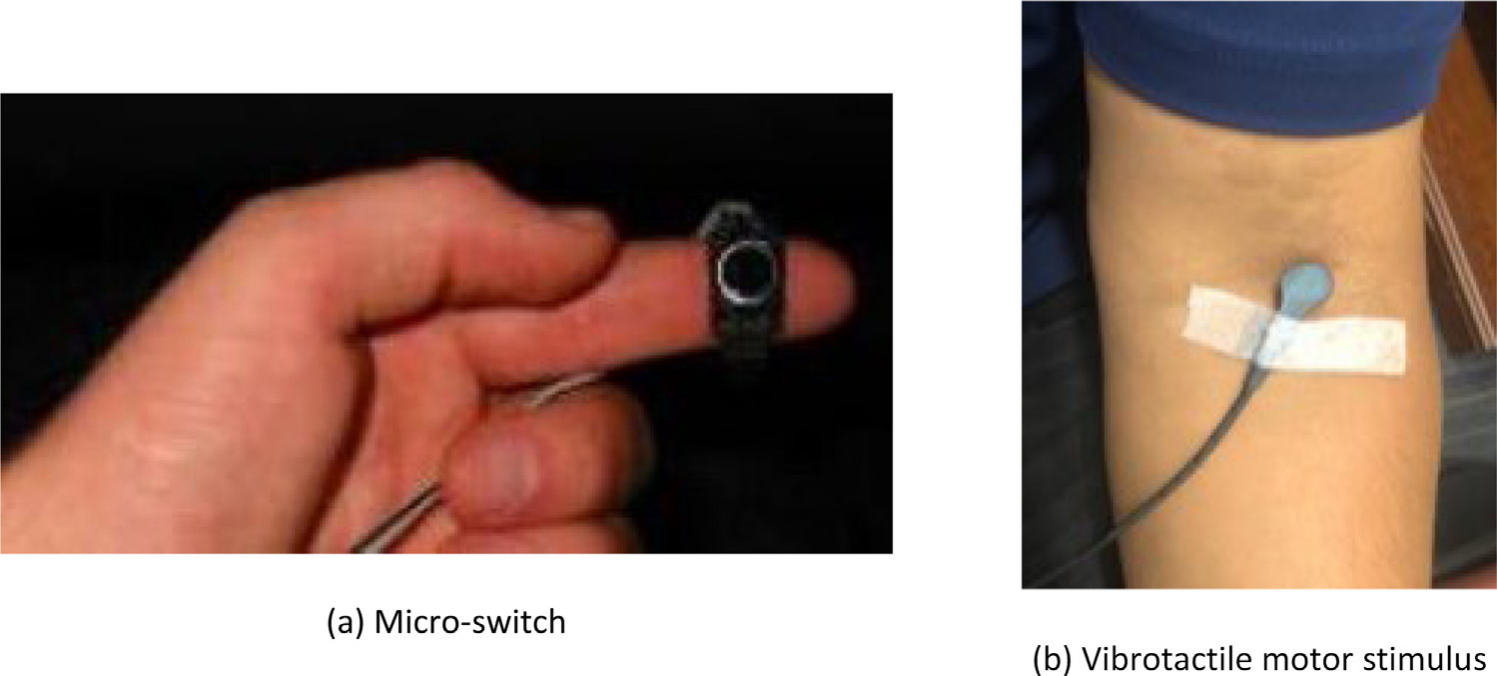
DRT. Vibrotactile version of the DRT used in the experiment.

A vibrotactile version of the DRT manufactured by Red Scientific LTD (Salt Lake City, Utah, United States) as per ISO 17488 (2016) was used in this experiment. A vibrotactile motor was placed on the participants’ left arm and a microswitch was attached to either the index or middle finger of the right hand, as in Fig. 8. Upon the presentation of a short stimulus similar to a phone vibration that occurred every 3 to 5 s, participants were instructed to press the microswitch as fast as possible. The time interval between the onset of the vibrotactile stimulus and the depression of the microswitch known as the response time was recorded.

### 2.4 Eye-tracker

**Fig. 9:**
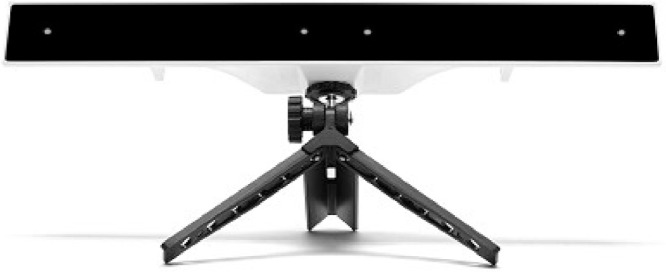
Eye-tracker. Gazepoint GP3 eye-tracker used in the experiment [6].

A desktop mounted eye-tracker (manufacturer and model name: Gazepoint GP3, see Fig. 9) was used to record the pupil diameter of the participants. Each time the data collection is preceded by a 9-point calibration phase where the participant is required to focus on the markers displayed on the screen. The eye-tracker provides a measure of pupil diameter in the number of pixels with a sampling rate of 60 Hz.

## Ethics Statement

All authors confirm that informed consent was obtained from each participant.

## Declaration of Competing Interest

The authors declare that they have no known competing financial interests or personal relationships which have, or could be perceived to have, influenced the work reported in this article.
